# Engineered *Pichia pastoris* production of fusaruside, a selective immunomodulator

**DOI:** 10.1186/s12896-019-0532-8

**Published:** 2019-06-17

**Authors:** Yuan Tian, Yanling Li, Fengchun Zhao, Chao Meng

**Affiliations:** 1 College of Life Science, Shandong First Medical University & Shandong Academy of Medical Sciences, Taian, 271016 Shandong China; 2 Department of Microbiology, College of Life Science, Key Laboratory for Agriculture Microbiology, Shandong Agricultural University, Taian, 271018 China

**Keywords:** Fusaruside, *P. pastoris*, Co-expression, Optimization

## Abstract

**Backgroud:**

Fusaruside is an immunomodulatory fungal sphingolipid which has medical potentials for treating colitis and liver injury, but its poor natural abundance limits its further study.

**Results:**

In this study, we described a synthetic biology approach for fusaruside production by engineered *Pichia pastoris* that was based on polycistronic expression. Two fusaruside biosynthesis genes (*Δ3(E)-sd* and *Δ10(E)-sd*), were introduced into *P. pastoris* to obtain fusaruside producing strain FUS2. To further enhance the yield of fusaruside, three relevant biosynthetic genes (*Δ3(E)-sd*, *Δ10(E)-sd* and *gcs*) were subsequently introduced into *P. pastoris* to obtain FUS3. All of the biosynthetic genes were successfully co-expressed in FUS2 and FUS3. Compared to that produced by FUS2, fusaruside achieved from FUS3 were slightly increased. In addition, the culture conditions including pH, temperature and methanol concentration were optimized to improve the fusaruside production level.

**Conclusions:**

Here a novel *P. pastoris* fusaruside production system was developed by introducing the biosynthetic genes linked by 2A peptide gene sequences into a polycistronic expression construct, laying a foundation for further development and application of fusaruside.

**Electronic supplementary material:**

The online version of this article (10.1186/s12896-019-0532-8) contains supplementary material, which is available to authorized users.

## Background

Fusaruside is a kind of sphingolipid, isolated as a minor compound from *Fusarium* endophytes [1, 2]. It has selective immunosuppression function, and is effective in treating T-cell-mediated colitis and liver injury via adjusting STAT1 signal pathway [3–5]. However, a more detailed study of the effects of fusaruside intake on animal and human health requires significant quantities of pure compounds for dietary research. The natural products for such feeding studies have traditionally been derived from fungal extracts or full chemical synthesis. In the case of fusaruside, such preparation methods may have problems. On the one hand, there are many closely related compounds in the extract mixture of *Fusarium* sp., on the other hand fusaruside are trace quantity in natrual fungi. Only 24 mg fusaruside could be obtained from about 200 g crude ethyl acetate extract [6]. Additionally, chemcal synthesis of fusaruside is tedious, inefficient and may cause environmental pollution [7]. As an alternative, we are interested in reconstructing the sphingolipid pathway from *Fusarium* into metabolic engineered *Pichia pastoris*, to produce a large number of clinically useful fusaruside.

**Fig. 1:**
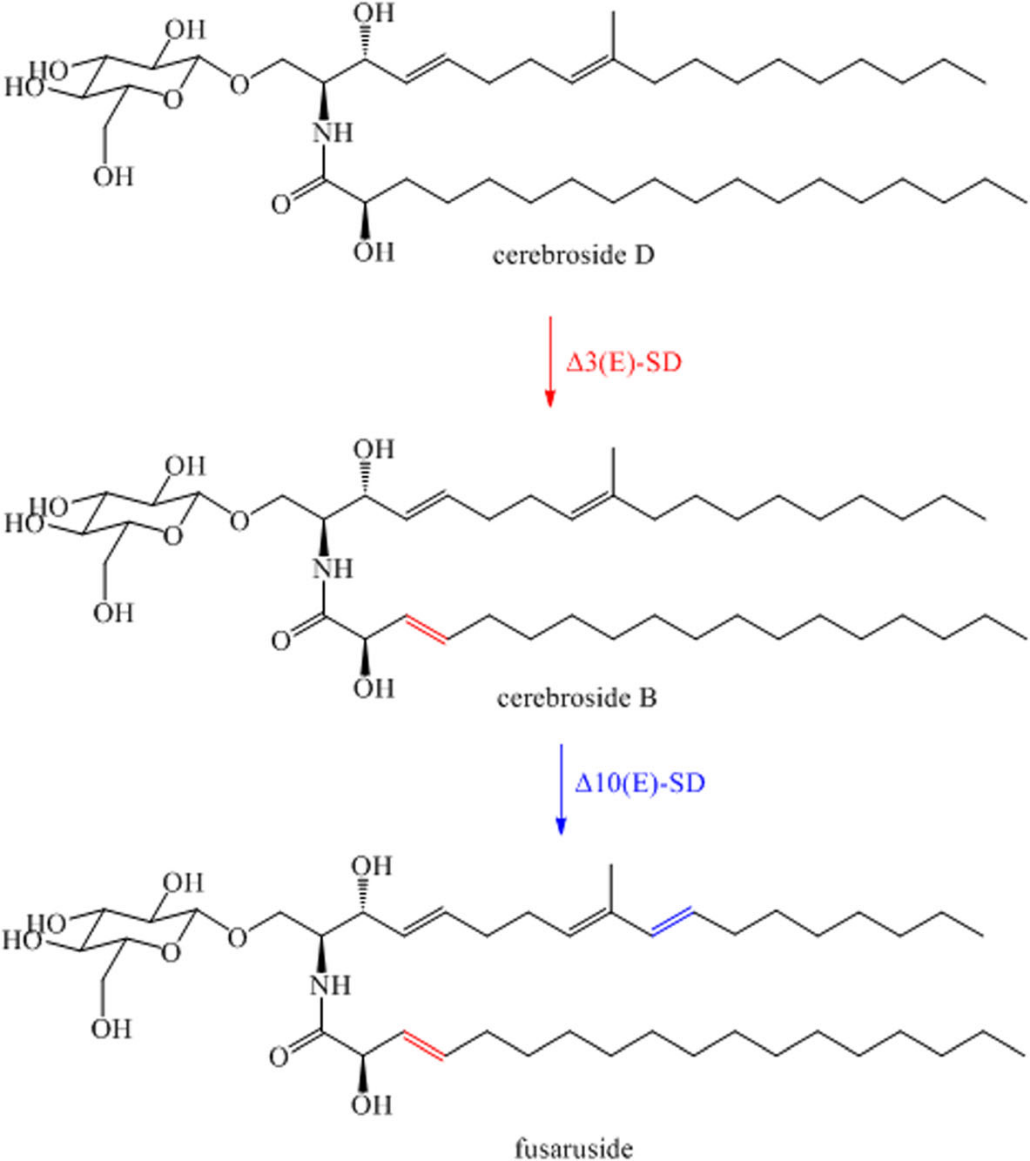
Engineered fusaruside biosynthetic pathway in *P. pastoris.* The engineered pathway includes the fusaruside biosynthetic proteins Δ3 (E)-SD and Δ10(E)-SD from *F. graminearum*

Based on early study, *P. pastoris* harbours cerebroside D [8] that differs from the precursor of fusaruside, cerebroside B. The only difference to cerebroside B from *F. graminearum* is the missing of C3-double bond on the N-Acyl chain. Cerebroside D can be metabolized by 2-hydroxy fatty N-acyl-delta3(E)-desaturase (Δ3(E)-SD) to form cerebroside B [9], and further converted into fusaruside in the presence of delta 10(E)-sphingolipid desaturase (Δ10(E)-SD) [10]. As cerebroside D exists in *P. pastoris*, it is highly plausible to target fusaruside biosynthesis through metabolic engineering of the yeast by co-overexpressing Δ10(E)-SD and Δ3(E)-SD (Fig. 1). Moreover, there are two separate pathways of sphingolipid biosynthesis in *P. pastoris* [8], and cerebroside D can be only produced by one of them. It has been proved that overexpressing glucosylceramide synthase (GCS) using the strong *AOX1* promoter could enhance the production of cerebroside D.

In this study, we firstly co-expressed Δ10(E)-SD and Δ3(E)-SD in yeast expression host systems to obtain fusaruside producing strain, then we co-expressed GCS with Δ10(E)-SD and Δ3(E)-SD to enhance the yield of fusaruside. The strategy adopted was to engineer yeast using a self-cleaving 2A peptide based vector system to realize synchronous production of the enzymes.

## Methods

### Chemicals and media

Restriction enzymes *Sac* I, *SnaB* I and *Not* I were purchased from Takara Bio Inc., Japan, and used as detailed by the manufacturer. Yeast nitrogen base w/o amino acids (YNB), D-sorbitol, Kanamycin monosulfate, Zeocin and geneticin sulfate (G-418S) were obtained from Solarbio, Beijing, China. Oxoid™ peptone and yeast extract were purchased from Thermo Scientific, Germany.

*P. pastoris* was grown in yeast peptone dextrose medium (YPD, 1% yeast extract, 2% peptone and 2% dextrose) or buffered complex glycerol medium (BMGY, 1% yeast extract, 2% peptone, 100 mM potassium phosphate, pH 6.0, 1.34% yeast nitrogen base, 0.4 mg/mL biotin, 1% glycerol). *P. pastoris* was induced in buffered complex methanol medium (BMMY, 1% yeast extract, 2% peptone, 100 mM potassium phosphate, pH 6.0, 1.34% yeast nitrogen base, 0.4 mg/mL biotin, 0.5% methanol). YPD plates containing 1 M Sorbitol and 2.5 mg/mL geneticin sulfate were used for selection of positive strains containing the pPIC3.5 K expression vector. *E. coli* was cultivated in LB medium (0.5% yeast extract, 1% peptone, 1% NaCl). *F. graminearum* was also grown in YPD medium.

### RNA isolation

For RNA isolation, *F. graminearum* PH-1 (NRRL 31084) was cultured on YPD medium for 72 h at 28 °C with shaking of 150 rpm. About 3 to 5 g wet fungal mycelium was collected by filtration on sterile filter paper and used for RNA extraction with the RNAeasy Mini Kit (Qiagen) following the manufacturer’s recommendations.

*P. pastoris* GS115 was grown on YPD medium for 24 h at 30 **°**C and 220 rpm. The yeast cells were collected by centrifugation with 5000 rpm and 5 to 10 g (wet weight) was used for RNA extraction using the RNAeasy Mini Kit.

### Gene amplification and synthesis

The cDNA was obtained by RT-PCR from total RNA, using PrimeScript™ 1st Strand cDNA Synthesis Kit (TaKaRa). The DNA fragment encoding the *F. graminearum Δ3(E)-sd* gene (GenBank accession: XM_383758.1) was amplified from cDNA by PCR using primers delta3-F (5′-*TACGTA*GCCACCATGGCCGAACACCTCGTCTTC) and delta3-R (5′-CTGCCTCTTAAACTTCTTC) which contain restriction sites (italicized) for *SnaB* I.

The *F. graminearum Δ10(E)-sd* gene (GenBank accession: XP_390021.1) was amplified by PCR using primers delta10-F (5′-ATGGCGCATAGCTCTTTCGTT) and delta10-R1 (5′-*GCGGCCGC*CTAGTGATGAGAGAGATCACC, *Not* I site is italicized) to co-express Δ3(E)-SD and Δ10(E)-SD, or amplified using primers delta10-F and delta10-R2 (5′-GTGATGAGAGAGATCACC) to co-express three enzymes, Δ3(E)-SD, Δ10(E)-SD and GCS. The *P. pastoris gcs* gene (GenBank accession: AF091397) was amplified using primers gcs-F (5′-ATGTCACAACTCAGACCCAG) and gcs-R (5′-*GCGGCCGC*TTACACTTCAAACCATGA, *Not*I site is italicized). The 2A polypeptide from the aphthovirus foot-and-month disease virus (FMDV) was selected and synthesized as part of a 66 bp synthetic sequence (5′-GGATCCGGAGCCACGAACTTCTCTCTGTTAAAGCAAGCAGGAGACGTGGAAGAAAACCCCGGTCCT) and cloned into pUC19 by BioSune Inc. (Shanghai).

PCR was performed with PrimeSTAR Max DNA Polymerase (TaKaRa) under the following cycling parameters: 30 s at 98 °C for a first denaturation step, 30 cycles of 10 s at 98 °C, 15 s at 55 °C and 30 s at 72 °C, and 1 cycle of a final extension step at 72 °C for 5 min. Reaction mixtures contained 1× PrimeSTAR Max Premix buffer (including Mg^2+^, dNTP and polymerase), 0.25 μM each primers and 10 ng template in a final volume of 50 μL.

### Construction of co-expression plasmid

**Fig. 2:**
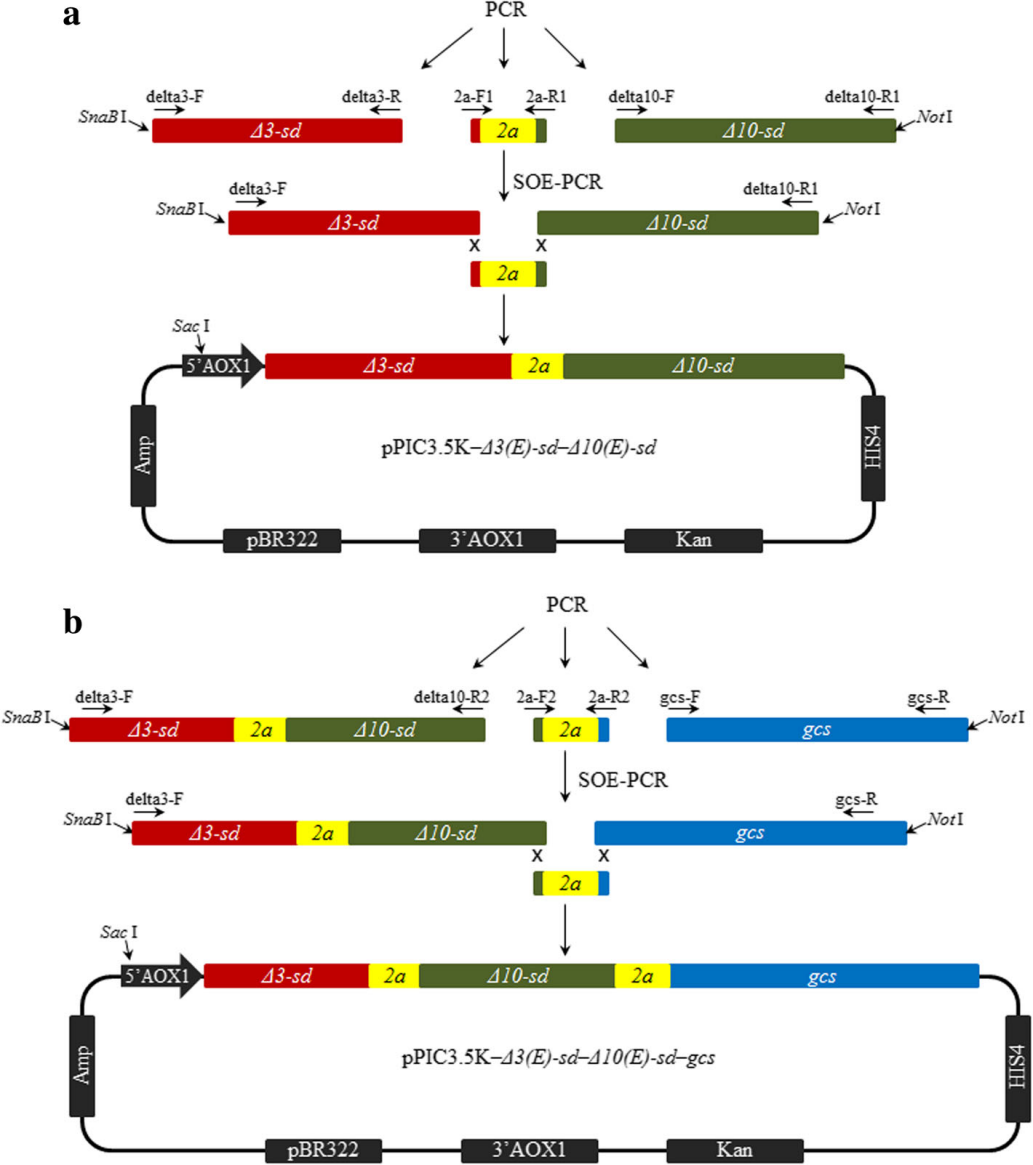
Schematic diagram showing the strategy for construction of the expression vector pPIC3.5 K*–Δ3(E)-sd–Δ10(E)-sd* (**a**) and pPIC3.5 K*–Δ3(E)-sd–Δ10(E)-sd–gcs* (**b**). Vector construction is described in “Materials and methods”

The strategy used to construct the plasmids for co-expression of the proteins described in this study is illustrated in Fig. 2.

To construct two enzymes co-expression vector, the genes *Δ3(E)-sd*, *Δ10(E)-sd* and *2a* were firstly amplified with delta3-F/delta3-R, delta10-F/delta10-R1 and 2a-F1 (5′- GAAGAAGTTTAAGAGGCAGGGATCCGGAGCCACGAAC)/2a-R1(CGAAAGAGCTATGCGCCATAGGACCGGGGTTTTCTTC) primers, respectively. The products were then fused by splicing overlap extension PCR (SOE-PCR) using delta3-F/delta10-R1 as primer. Finally, the fused gene *Δ3(E)-sd–2a–Δ10(E)-sd* were sub-cloned into pPIC3.5 K, using *SnaB* I and *Not* I sites (Fig. 2a).

To construct three proteins co-expression plasmid, the fused gene *Δ3(E)-sd–2a–Δ10(E)-sd*, *gcs* and *2a* were amplified with delta3-F/delta10-R2, gcs-F/gcs-R and 2a-F2 (5′-GGTGATCTCTCTCATCACGGATCCGGAGCCACGAAC)/2a-R2(5′-GGGTCTGAGTTGTGACATAGGACCGGGGTTTTCTTC) primers, respectively. Subsequently, the products were fused by SOE-PCR using delta3-F/gcs-R as primer. The fused products *Δ3(E)-sd–2a–Δ10(E)-sd–2a–gcs* were finally sub-cloned into pPIC3.5 K, using *SnaB* I and *Not* I sites (Fig. 2b).

The recombinant plasmids were transformed into *E. coli* DH5α, and positive transformants were identified by restriction digest analysis and sequencing.

### Transformation of *P. pastoris*

To obtain stable expression strains, the genes in expression vectors (including 5’AOX1, gene of insert, HIS4, Kan and 3’AOX1) were usually transformed to *P. pastoris* and integrated into the genome [11]. The procedure of transformation was performed according to the protocol of Lin-Cereghino [12]. Firstly, the expression vector was linearized with *Sac* I for integration into the genome of *P. pastoris*. Subsequently, 2 μg of linearized plasmids and competent *P. pastoris* cells were mixed thoroughly and transferred to ice-cold electroporation cuvettes (0.2 cm; Bio-Rad, America). The electroporational parameters were set as 200 Ω, 25 μF and 1.5 kV. After pulse, 500 μL of ice-cold 1 M sorbitol was added immediately and incubated at 28 °C for 1 h. After that, another 500 μL of YPD was added for further 2 h of regeneration at the same temperature. After 5~7 days, positive transformants were selected on YPD plates containing 2.5 mg/mL Geneticin 418, and then lysed using a combination of enzyme, freezing and heating according to a simple protocol described in the literature [13]. The cell lysates that contain the genomic DNA were analyzed by PCR using the primers delta3-F/delta10-R1 or delta3-F/gcs-R. Linearized vector pPIC3.5 K was also transformed into *P. pastoris* to be used as negative control.

### Expression of 2A polyprotein constructs in *P. pastoris* yeast

Positive clones were cultured in 10 mL BMGY medium in a 100 mL flask at 30 °C with shaking of 200 rpm until OD600 reaching 12~16. At room temperature, the cultures were collected by centrifugation at 5000 rpm for 5 min. The cell pellets were suspended in 20 mL BMMY medium and grew under the same conditions (30 °C, 200 rpm). Methanol was added to the culture every 24 h to a final concentration of 0.5% (v/v) to induce proteins co-expression. At the same time, 1 mL of culture medium was collected every 24 h after initiating induction. The yeast cells were immediately frozen and stored for further sodium dodecyl sulfate-polyacrylamide gelelectrophoresis (SDS-PAGE) analysis. The theoretical molecular weights (MW) of the biosynthetic enzymes were calculated using the website http://web.expasy.org/compute_pi/.

### Isolation of sphingolipids

After 84 h of induction, *P. pastoris* cells were treated as described [9]. Approximately 50 mg of dried *P. pastoris* cells were firstly suspended in 5 mL of H_2_O and boiled in a water bath for 15 min. Then, the cells were sedimented by centrifugation at 5000 rpm for 5 min and used to extract sphingolipids. After that, 10 mL of dichloromethane/methanol (v/v = 1:1) was added and shaken overnight at 4 °C to extract the yeast sphingolipids. The next day, another 10 mL of dichloromethane/methanol (v/v = 2:1) was added and shaken for at least 4 h at 4 °C. Subsequently, the 20 mL of sphingolipid extract was washed three times with dichloromethane/methanol/0.45% NaCl (v/v = 8:4:3). The organic phase (dichloromethane, in the lower layer) was collected and the solvents were removed by a rotary evaporator.

### LC-MS analysis and isolation of sphingolipids

Sphingolipids extracts in organic solvents were analyzed and quantified by liquid chromatography technique coupled with mass spectrometry (LC-MS) as our previous study [10]. To identifiy the compounds in the extract mixture, reference standards (fusaruside and cerebroside B) were used and the derived mass fragmentation spectra were compared. To confirm the structure of fusaruside, sphingolipids extracts were isolated by chromatography and determined by nuclear magnetic resonance (NMR) analysis as described [10].

### Optimization of fusaruside production

In order to increase the production of fusaruside in yeast, culture and induction conditions of engineered *P. pastoris* including methanol concentration, pH and temperature were optimized. All experiments were performed in triplicates.

## Results

### Construction of 2A polyprotein cassettes

Two polyprotein cassettes consisting of *Δ3(E)-sd* and *Δ10(E)-sd* genes, or consisting of *Δ3(E)-sd*, *Δ10(E)-sd* and *gcs* genes, separated by the 2A sequences were cloned into the *P. pastoris* expression vector pPIC3.5 K, which is under the regulation of the *AOX1* promoter (Fig. 2). The plasmids were further transformed into *E. coli* DH5α to amplify and confirm (see Additional file 1: Figure S1). Double enzyme digestion and sequencing indicated that the genes were correctly oriented in the pPIC3.5 K vector. The theoretical MW of Δ3(E)-SD, Δ10(E)-SD and GCS were 50 kDa, 65 kDa and 133 kDa, respectively.

### Transformation and screening of transformants

The plasmids pPIC3.5 K − *Δ3(E)-sd* − *Δ10(E)-sd* and pPIC3.5 K − *Δ3(E)-sd* − *Δ10(E)-sd* − *gcs* were linearized with *Sac* I and transformed into *P. pastoris* GS115 competent cells by electroporation, respectively. Positive transformants were selected by the ability to grow on YPD plates containing 2.5 mg/mL of Geneticin 418, due to the presence of *Kan* gene in the pPIC3.5 K vector. The genomic DNA of *P. pastoris* recombinants was isolated to perform PCR verification using delta3-F/delta10-R1 or delta3-F/gcs-R as primers. One band corresponding to the size of the *Δ3(E)-sd* gene plus the *Δ10(E)-sd* gene (~ 3000 bp) were obtained in the chromosome of *P. pastoris* intergrated by pPIC3.5 K − *Δ3(E)-sd* − *Δ10(E)-sd* (see Additional file 1: Figure S2a). In the other *P. pastoris* recombinant containing pPIC3.5 K − *Δ3(E)-sd* − *Δ10(E)-sd* − *gcs*, a band corresponding to the size of three genes (~ 6000 bp) were also detected (see Additional file 1: Figure S2b).

### Expression of polycistronic constructs encoding fusaruside pathway

**Fig. 3:**
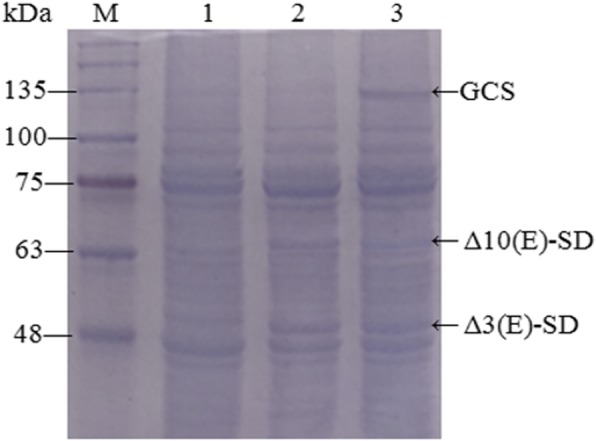
SDS-PAGE analysis of proteins in recombinantyeasts induced with 0.5% methanol for 96 h. M: protein molecular weight marker (Solarbio); lane 1: pPIC3.5 K (negative control); lane 2: clone FUS2; lane 3: clone FUS3. The position of the bands corresponding to Δ3(E)-SD, Δ10(E)-SD and GCS is indicated by *arrows*

Two clones named FUS2 and FUS3 were selected to detect enzymes co-expression under inductive conditions. Yeast cells collected after a 4-day induction with methanol were analyzed by SDS-PAGE. The cells from clone FUS2 showed two major induction bands at approximately 50 kDa and 65 kDa (Fig. 3, lane 2), and three target bands at approximately 50 kDa, 65 kDa and 133 kDa were observed in FUS3 cells (Fig. 3, lane 3). A yeast strain transformed with pPIC3.5 K was used as control (Fig. 3, lane 1), and no target band was detected in the cells of an induced transformant harboring an empty plasmid. The results thus confirming that the fusaruside biosynthetic enzymes had been successfully co-expressed.

### Analysis of sphingolipids in *P. pastoris* strains

**Fig. 4:**
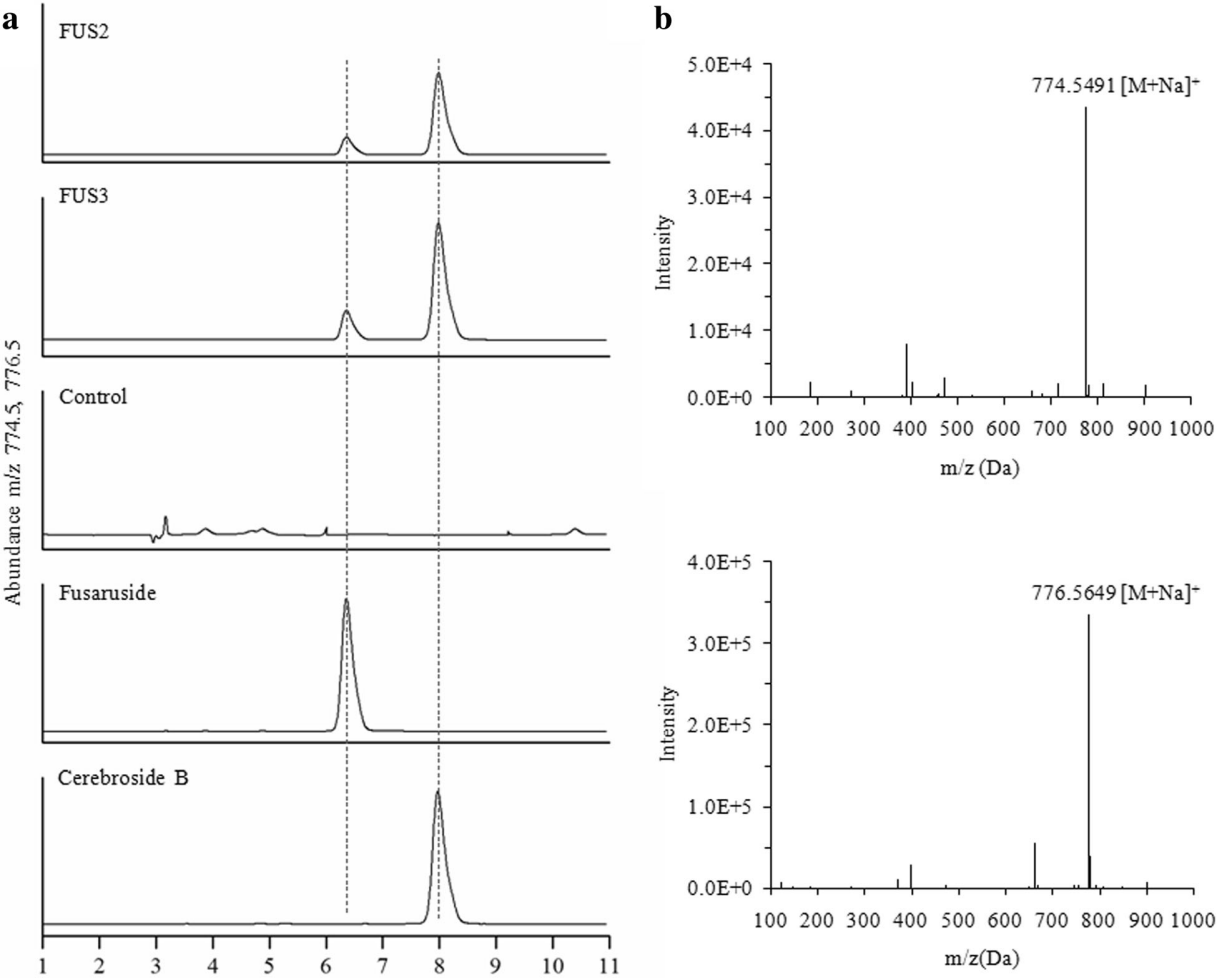
LC-MS analysis of the sphingolipids from recombinant *P. pastoris* strains FUS2 (pPIC3.5 K − *Δ3(E)-sd* − *Δ10(E)-sd*), FUS3 (pPIC3.5 K − *Δ3(E)-sd* − *Δ10(E)-sd − gcs*) and negative control (pPIC3.5 K). Shown are representative chromatograms. **a** Detection of ion m/z = 774.5 and 776.5 representative of fusaruside and cerebroside B in FUS2, FUS3 and negative control strains, and in a 5 μg/mL standard solution, respectively. **b** MS spectra of fusaruside and cerebroside B, corresponding to the Na + −liganded molecular ions at m/z 774.5491 (774.5491 calcd. For C_43_H_77_NO_9_Na) and 776.5649 (776.5649 calcd. For C_43_H_79_NO_9_Na)

Sphingolipids extracted from the *P. pastoris* transformants were analyed by LC-MS. As displayed in Fig. 4, the co-expression of the desaturases Δ3(E)-SD and Δ10(E)-SD from *F. graminearum* led to the formation of unsaturated fatty acids, cerebroside B and fusaruside. Compared with that in the strain FUS2 carrying two genes, the yield of fusaruside in FUS3 harboring three genes was slightly increased. While sphingolipids extracted from negative control, *P. pastoris* transformed with pPIC3.5 K, produced no cerebroside B or fusaruside. The sphingolipids production was quantifed by LC-MS. Strain FUS2, which co-expressed two enzymes, produced 1.01 ± 0.17 mg/g (mg of product per gram of sphingolipids crude extracts) cerebroside B and 0.39 ± 0.04 mg/g fusaruside. The cerebroside B and fusaruside produced by strain FUS3 were 1.25 ± 0.22 and 0.52 ± 0.06 mg/g, respectively. The identity of cerebroside B and fusaruside isolated from *P. pastoris* transformants were also confirmed by NMR spectroscopy. The ^1^H NMR spectra of cerebroside B (see Additional file 1: Figure S3) and fusaruside (see Additional file 1: Figure S4) were identical to those of authentic materials [9, 10].

### Optimization of growth and induction

Three different culture conditions including pH, temperature and methanol concentration were optimized to improve fusaruside production in shake flask, due to their importance for *P. pastoris* expression system [14, 15]. After 120 h of induction under different conditions, all the biomass concentrations (OD600) could reach to maximum (~ 8).

**Fig. 5:**
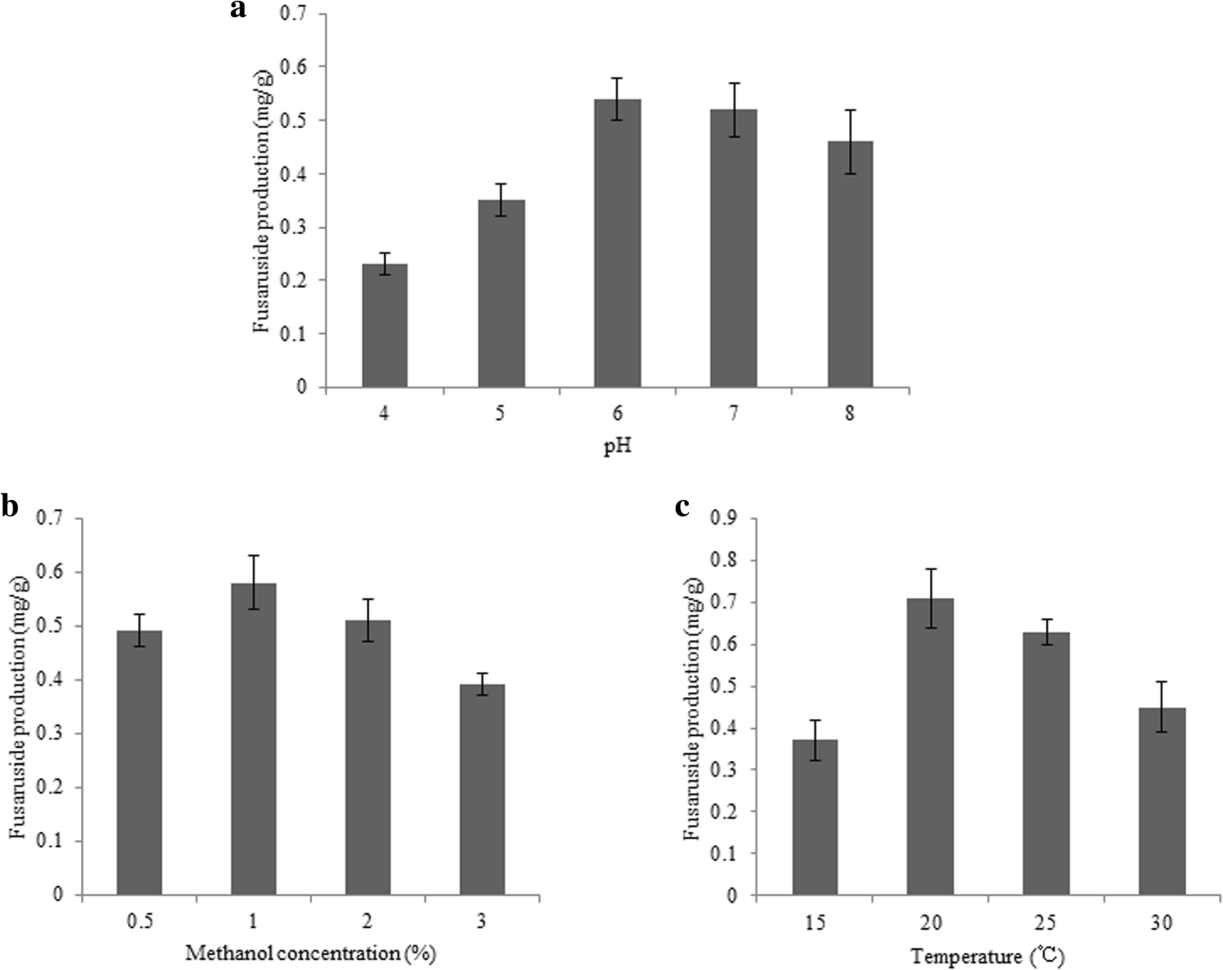
Optimization of fusaruside production. Effect of pH (**a**), different methanol concentration (**b**) and temperature (**c**) on the production of fusaruside in ecombinant *P. pastoris* strain FUS3

The strain of FUS3, co-expressing three enzymes, was investigated in BMMY media at different pH levels (4.0, 5.0, 6.0, 7.0 and 8.0). As shown in Fig. 5a, the maximum yield of fusaruside was observed at pH 6.0. In order to optimize methanol concentration, various final concentrations of methanol (0.5, 1, 2 and 3%) was applied to induce enzyme co-expression in BMMY. The results in Fig. 5b demonstrated that the best methanol concentration for fusaruside production was 1%. Finally, FUS3 was cultivated at 15, 20, 25 and 30 °C in order to find the optimum temperature for fusaruside production. The results in Fig. 5c indicated that highest yield was reached at 20 °C. After optimization, high level (0.74 ± 0.08 mg/g) of fusaruside was obtained in engineered *P. pastoris* FUS3 strain after 120 h induction at optimum condition (pH 6.0, 1% methanol concentration and 20 °C).

## Discussions

Fusaruside is a potential pharmaceutical molecule due to its ability to treat liver injury and colitis through selective immunosuppressive effect. But its poor natural abundance in *Fusarium* sp. endophytes has become the main bottleneck restricting its further pharmacological research. In this work we sought to produce fusaruside in engineered *P. pastoris* by co-expressing biosynthetic genes, Δ3(E)-SD, Δ10(E)-SD and GCS. A self-processing 2A peptide from FMDV was applied to construct polycistronic systems for gene co-expression in yeast, due to its capacity of simultaneous and efficient expression of multiple genes [16–19]. 2A self-cleavage peptides has been extensively exploited in biomedicine and biotechnology. E.g. A flavone-C-glycoside pathway was reconstructed in tobacco and yeast using 2A self-cleavage peptide [20]. Similarly, an entire penicillin biosynthesis pathway was rebuilt in *Aspergillus nidulans* strains using 2A peptide-based system [21]. Geier et al. reported for the first time the functional simultaneous expression of nine genes from a single 2A peptide based polycistronic expression construct to produce violacein and carotenoid in *P. pastoris* [22].

Although the individual expression of Δ3(E)-SD and Δ10(E)-SD in *P. pastoris* has been previously described [9, 10]. We coexpressed the two enzymes succesfully to realize the production of fusaruside in yeast. Two prominent protein bands representing the two enzymes were produced by clone FUS2 (theoretically in a 1:1 ratio) which is consistent with their co-translational production. LC-MS indicated that the yield of fusaruside produced by FUS2 was 0.39 ± 0.04 mg/g. To increase the yield further, GCS was also co-expressed with the two desaturases in strain FUS3, because of its importance for sphingolipid biosynthesis in *P. pastoris* [8]. As we expected, the yield of fusaruside in FUS3 increased to 0.52 ± 0.06 mg/g.

It has been demonstrated that growth and induction conditions are critical parameters for *P. pastoris* expression systerm [23–25], thus the conditions of FUS3 producing fusaruside were optimized. In this study the optimum pH, methanol concentration and temperature were investigated. pH may affect the yield of fusaruside through influencing the recombinant enzyme activity. As observed in Fig. 5a, although fusaruside produced at all pH levels, the maximum yield was at pH 6.0. Due to the use of inducible *AOX1* promoter [14], methanol level can impact the expression of proteins and thus influence the production of fusaruside. A low concetration of methanol cannot induce the promoter efficiently, but excess methanol can lead to an increase of misfolded proteins [26–28]. Here the highest fusaruside production observed at 1% methanol concentration (Fig. 5b). According to many protocols, the optimum temperature for *P. pastoris* cell growth is 30 °C. But in our previous study, the content of fusaruside may increase at lower temperature to resistant cold environment [10]. We asked if lower temperature could enhance the yield of fusaruside in the engineered yeast. When analysing the sphingolipids of FUS3 after 120 h of induction, the maximum production was observed at 20 °C (Fig. 5c). The growth of *Pichia* cells declined too much at 15 °C, and therefore fusaruside production was affected. Finally, at optimum condition (pH 6.0, 20 °C and 1% methanol concentration) a high level (0.74 ± 0.14 mg/g) of fusaruside in yeast FUS3 was achieved after 120 h induction. Compared to the yield of original *Fusarium* sp. (0.12 mg/g after 10 d) [1], the production in *P. pastoris* was satisfactory.

To further improve the yield of fusaruside, there are still several measures can be done. Except the 2A sequence from FMDV used in this work, there are some other 2A sequences, such asthe P2A sequence from porcine teschovirus-1 and T2A from *Thosea asigna* virus [29]. It has been reported that T2A functioned better than other 2A sequences and was used to reconstruct carotene biosynthetic pathway in *Saccharomyces cerevisiae* [30]. Thus different 2A sequences can be considered in further study to enhance fusaruside production. A recent literature indicated that the alteration of the order of genes in the polycistronic 2A construct would impact the pathway, and then affect the production of metabolites [31]. This gives us a reminder that the possibility of improving output by changing the order of *Δ3(E)-sd*, *Δ10(E)-sd* and *gcs* genes.

## Conclusions

In summary, we reconstituted a heterologous fusaruside biosynthetic pathway by linking three genes, *Δ3(E)-sd*, *Δ10(E)-sd* and *gcs*, via 2A peptide sequences. Thus, the engineered *P. pastoris* yeast can generate fusaruside via glycosphingolipid pathway, and this opens for the use of yeast as a cell factory for production of cerebrosides in future.

## Additional file

Additional file 1: **Figure S1.** Identification of co-expression plasmid by digestion. **a**. M: DNA marker; 1: product of double enzyme digestion of pPIC3.5 K − *Δ3(E)-sd* − *Δ10(E)-sd*. **b**. M: DNA marker; 1: product of double enzyme digestion of pPIC3.5 K − *Δ3(E)-sd* − *Δ10(E)-sd* − gcs. **Figure S2.** Identification of transformants by PCR. **a**. M: DNA marker; 1–2: PCR products with primer delta3-F/ delta10-R. **b**. M: DNA marker; 1–2: PCR products with primer delta3-F/gcs-R. **Figure S3.** ^1^H NMR of cerebroside B in CDCl_3_ (400 MHz). **Figure S4.** ^1^H NMR of fusaruside in CDCl_3_ (400 MHz). (DOCX 341 kb)

## Data Availability

The datasets supporting the conclusions of this article are available from the corresponding author on reasonable request.

## Abbreviations

BMGY: Buffered complex glycerol medium
BMMY: Buffered complex methanol medium
FMDV: Foot-and-month disease virus
GCS: Glucosylceramide synthase
LC-MS: Liquid chromatography technique coupled with mass spectrometry
NMR: Nuclear magnetic resonance
SDS-PAGE: Sodium dodecyl sulfate-polyacrylamide gelelectrophoresis
YNB: Yeast nitrogen base w/o amino acids
YPD: Yeast peptone dextrose medium
Δ10(E)-SD: delta 10(E)-sphingolipid desaturase
Δ3(E)-SD: 2-hydroxy fatty N-acyl-delta3(E)-desaturase
