# Supplementary material for: Engineered Pichia pastoris production of fusaruside, a selective immunomodulator
Source: BMC Biotechnol. 2019 Jun 17;19:37. doi: 10.1186/s12896-019-0532-8 (PMC6580515; doi:10.1186/s12896-019-0532-8)
Supplement: Supplementary file 1 — Figure S1. Identification of co-expression plasmid by digestion. a. M: DNA marker; 1: product of double enzyme digestion of pPIC3.5 K − Δ3(E)-sd − Δ10(E)-sd. b. M: DNA marker; 1: product of double enzyme digestion of pPIC3.5 K − Δ3(E)-sd − Δ10(E)-sd − gcs. Figure S2. Identification of transformants by PCR. a. M: DNA marker; 1–2: PCR products with primer delta3-F/ delta10-R. b. M: DNA marker; 1–2: PCR products with primer delta3-F/gcs-R. Figure S3. 1H NMR of cerebroside B in CDCl3 (400 MHz). Figure S4. 1H NMR of fusaruside in CDCl3 (400 MHz). (DOCX 341 kb) [file 12896_2019_532_MOESM1_ESM.docx]

Engineered*Pichia pastoris* production of fusaruside, a selective immunomodulator

Yuan Tian*^,1^, Yanling Li^1^, Fengchun Zhao^2^ and Chao Meng^1^.

1. College of Life Science, Shandong First Medical University & Shandong Academy of Medical Sciences, Taian 271016, China

2. Department of Microbiology, College of Life Science, Key Laboratory for Agriculture Microbiology, Shandong Agricultural University, Taian 271018, China

Yuan Tian,Yanling Li and Fengchun Zhao equally contributed in this study.

*Correspondence: Email: tianyuan2005hit@163.com; Tel.: +86-358-623-5778


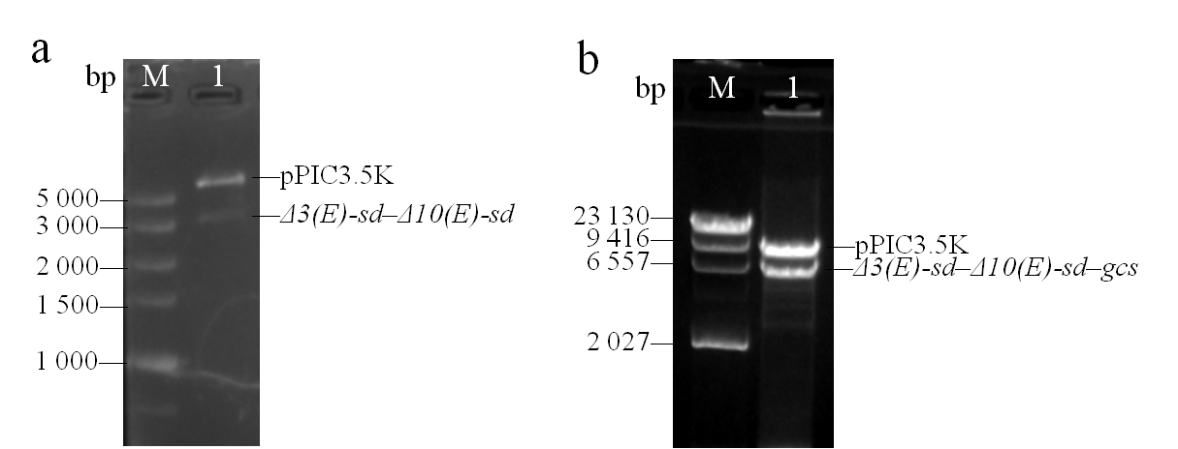


**Fig.S1** Identification of co-expression plasmid by digestion. **a**. M: DNA marker;1: product of double enzyme digestion of pPIC3.5K−*Δ3(E)-sd*−*Δ10(E)-sd*. **b**. M: DNA marker;1: product of double enzyme digestion of pPIC3.5K−*Δ3(E)-sd*−*Δ10(E)-sd*−*gcs*.

**
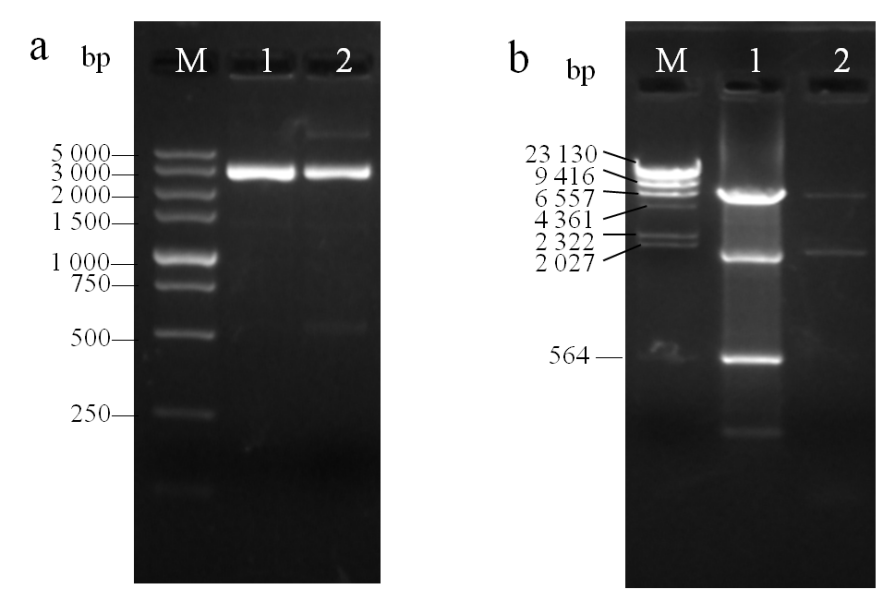
**

**Fig. S2** Identification of transformants by PCR. **a**. M: DNA marker; 1-2: PCR products with primer delta3-F/delta10-R. **b**. M: DNA marker; 1-2: PCR products with primer delta3-F/gcs-R.

**
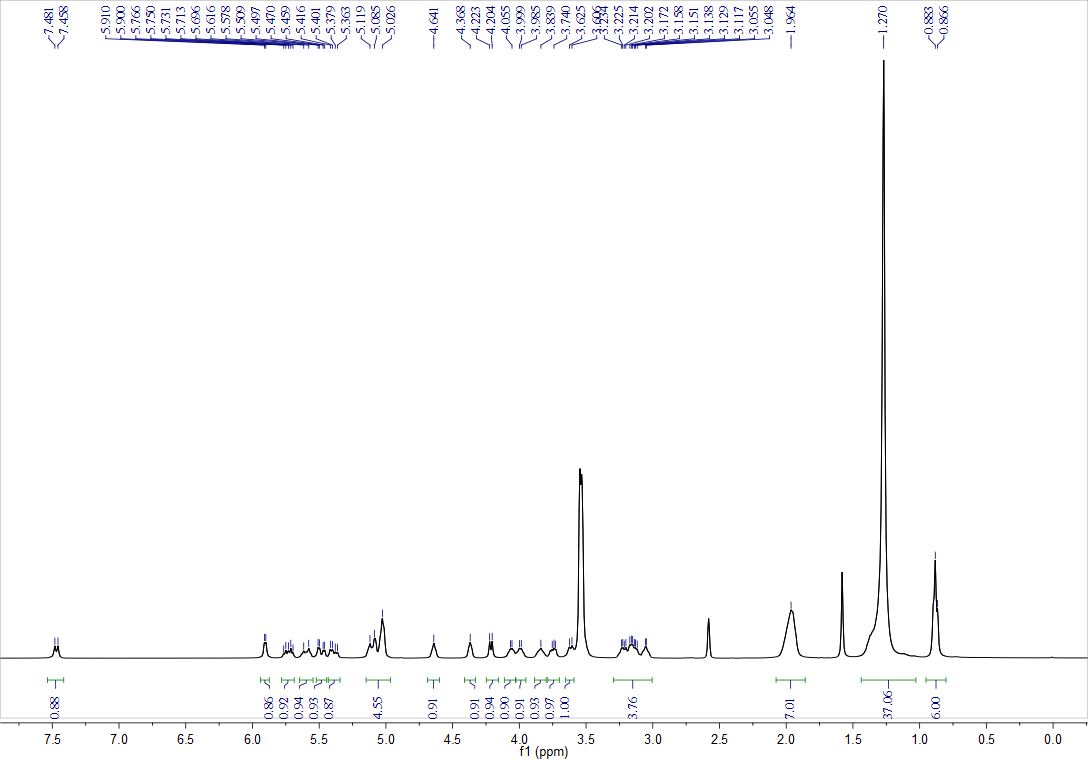
**

Fig. S3 ^1^HNMR of cerebroside B in DMSO-d_6_ (400 MHz).

**
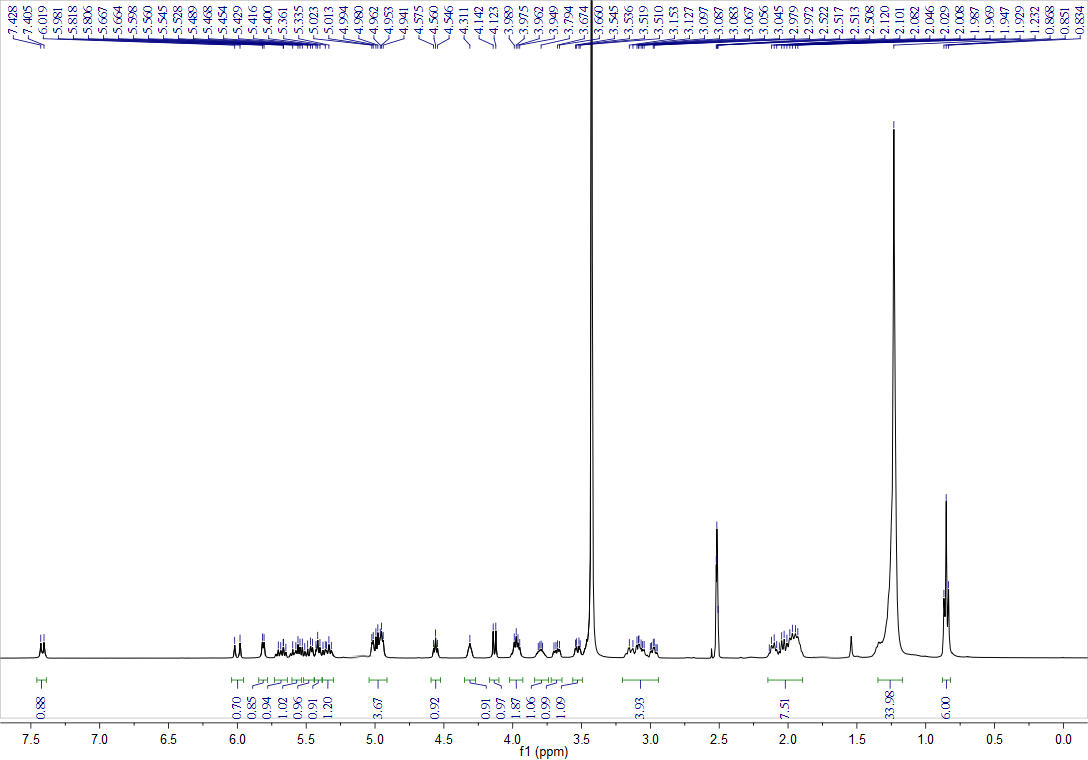
**

Fig. S4 ^1^HNMR of fusaruside in DMSO-d_6_ (400 MHz).

The full sequence of plasmid pPIC3.5K.

AGATCTAACATCCAAAGACGAAAGGTTGAATGAAACCTTTTTGCCATCCGACATCCACAG

GTCCATTCTCACACATAAGTGCCAAACGCAACAGGAGGGGATACACTAGCAGCAGACCGT

TGCAAACGCAGGACCTCCACTCCTCTTCTCCTCAACACCCACTTTTGCCATCGAAAAACC

AGCCCAGTTATTGGGCTTGATTGGAGCTCGCTCATTCCAATTCCTTCTATTAGGCTACTA

ACACCATGACTTTATTAGCCTGTCTATCCTGGCCCCCCTGGCGAGGTTCATGTTTGTTTA

TTTCCGAATGCAACAAGCTCCGCATTACACCCGAACATCACTCCAGATGAGGGCTTTCTG

AGTGTGGGGTCAAATAGTTTCATGTTCCCCAAATGGCCCAAAACTGACAGTTTAAACGCT

GTCTTGGAACCTAATATGACAAAAGCGTGATCTCATCCAAGATGAACTAAGTTTGGTTCG

TTGAAATGCTAACGGCCAGTTGGTCAAAAAGAAACTTCCAAAAGTCGGCATACCGTTTGT

CTTGTTTGGTATTGATTGACGAATGCTCAAAAATAATCTCATTAATGCTTAGCGCAGTCT

CTCTATCGCTTCTGAACCCCGGTGCACCTGTGCCGAAACGCAAATGGGGAAACACCCGCT

TTTTGGATGATTATGCATTGTCTCCACATTGTATGCTTCCAAGATTCTGGTGGGAATACT

GCTGATAGCCTAACGTTCATGATCAAAATTTAACTGTTCTAACCCCTACTTGACAGCAAT

ATATAAACAGAAGGAAGCTGCCCTGTCTTAAACCTTTTTTTTTATCATCATTATTAGCTT

ACTTTCATAATTGCGACTGGTTCCAATTGACAAGCTTTTGATTTTAACGACTTTTAACGA

CAACTTGAGAAGATCAAAAAACAACTAATTATTCGAAGGATCCTACGTAGAATTCCCTAG

GGCGGCCGCGAATTAATTCGCCTTAGACATGACTGTTCCTCAGTTCAAGTTGGGCACTTA

CGAGAAGACCGGTCTTGCTAGATTCTAATCAAGAGGATGTCAGAATGCCATTTGCCTGAG

AGATGCAGGCTTCATTTTTGATACTTTTTTATTTGTAACCTATATAGTATAGGATTTTTT

TTGTCATTTTGTTTCTTCTCGTACGAGCTTGCTCCTGATCAGCCTATCTCGCAGCTGATG

AATATCTTGTGGTAGGGGTTTGGGAAAATCATTCGAGTTTGATGTTTTTCTTGGTATTTC

CCACTCCTCTTCAGAGTACAGAAGATTAAGTGAGACGTTCGTTTGTGCAAGCTTATCGAT

AAGCTTTAATGCGGTAGTTTATCACAGTTAAATTGCTAACGCAGTCAGGCACCGTGTATG

AAATCTAACAATGCGCTCATCGTCATCCTCGGCACCGTCACCCTGGATGCTGTAGGCATA

GGCTTGGTTATGCCGGTACTGCCGGGCCTCTTGCGGGATATCGTCCATTCCGACAGCATC

GCCAGTCACTATGGCGTGCTGCTAGCGCTATATGCGTTGATGCAATTTCTATGCGCACCC

GTTCTCGGAGCACTGTCCGACCGCTTTGGCCGCCGCCCAGTCCTGCTCGCTTCGCTACTT

GGAGCCACTATCGACTACGCGATCATGGCGACCACACCCGTCCTGTGGATCTATCGAATC

TAAATGTAAGTTAAAATCTCTAAATAATTAAATAAGTCCCAGTTTCTCCATACGAACCTT

AACAGCATTGCGGTGAGCATCTAGACCTTCAACAGCAGCCAGATCCATCACTGCTTGGCC

AATATGTTTCAGTCCCTCAGGAGTTACGTCTTGTGAAGTGATGAACTTCTGGAAGGTTGC

AGTGTTAACTCCGCTGTATTGACGGGCATATCCGTACGTTGGCAAAGTGTGGTTGGTACC

GGAGGAGTAATCTCCACAACTCTCTGGAGAGTAGGCACCAACAAACACAGATCCAGCGTG

TTGTACTTGATCAACATAAGAAGAAGCATTCTCGATTTGCAGGATCAAGTGTTCAGGAGC

GTACTGATTGGACATTTCCAAAGCCTGCTCGTAGGTTGCAACCGATAGGGTTGTAGAGTG

TGCAATACACTTGCGTACAATTTCAACCCTTGGCAACTGCACAGCTTGGTTGTGAACAGC

ATCTTCAATTCTGGCAAGCTCCTTGTCTGTCATATCGACAGCCAACAGAATCACCTGGGA

ATCAATACCATGTTCAGCTTGAGACAGAAGGTCTGAGGCAACGAAATCTGGATCAGCGTA

TTTATCAGCAATAACTAGAACTTCAGAAGGCCCAGCAGGCATGTCAATACTACACAGGGC

TGATGTGTCATTTTGAACCATCATCTTGGCAGCAGTAACGAACTGGTTTCCTGGACCAAA

TATTTTGTCACACTTAGGAACAGTTTCTGTTCCGTAAGCCATAGCAGCTACTGCCTGGGC

GCCTCCTGCTAGCACGATACACTTAGCACCAACCTTGTGGGCAACGTAGATGACTTCTGG

GGTAAGGGTACCATCCTTCTTAGGTGGAGATGCAAAAACAATTTCTTTGCAACCAGCAAC

TTTGGCAGGAACACCCAGCATCAGGGAAGTGGAAGGCAGAATTGCGGTTCCACCAGGAAT

ATAGAGGCCAACTTTCTCAATAGGTCTTGCAAAACGAGAGCAGACTACACCAGGGCAAGT

CTCAACTTGCAACGTCTCCGTTAGTTGAGCTTCATGGAATTTCCTGACGTTATCTATAGA

GAGATCAATGGCTCTCTTAACGTTATCTGGCAATTGCATAAGTTCCTCTGGGAAAGGAGC

TTCTAACACAGGTGTCTTCAAAGCGACTCCATCAAACTTGGCAGTTAGTTCTAAAAGGGC

TTTGTCACCATTTTGACGAACATTGTCGACAATTGGTTTGACTAATTCCATAATCTGTTC

CGTTTTCTGGATAGGACGACGAAGGGCATCTTCAATTTCTTGTGAGGAGGCCTTAGAAAC

GTCAATTTTGCACAATTCAATACGACCTTCAGAAGGGACTTCTTTAGGTTTGGATTCTTC

TTTAGGTTGTTCCTTGGTGTATCCTGGCTTGGCATCTCCTTTCCTTCTAGTGACCTTTAG

GGACTTCATATCCAGGTTTCTCTCCACCTCGTCCAACGTCACACCGTACTTGGCACATCT

AACTAATGCAAAATAAAATAAGTCAGCACATTCCCAGGCTATATCTTCCTTGGATTTAGC

TTCTGCAAGTTCATCAGCTTCCTCCCTAATTTTAGCGTTCAACAAAACTTCGTCGTCAAA

TAACCGTTTGGTATAAGAACCTTCTGGAGCATTGCTCTTACGATCCCACAAGGTGGCTTC

CATGGCTCTAAGACCCTTTGATTGGCCAAAACAGGAAGTGCGTTCCAAGTGACAGAAACC

AACACCTGTTTGTTCAACCACAAATTTCAAGCAGTCTCCATCACAATCCAATTCGATACC

CAGCAACTTTTGAGTTGCTCCAGATGTAGCACCTTTATACCACAAACCGTGACGACGAGA

TTGGTAGACTCCAGTTTGTGTCCTTATAGCCTCCGGAATAGACTTTTTGGACGAGTACACCAGGCCCAACGAGTAATTAGAAGAGTCAGCCACCAAAGTAGTGAATAGACCATCGGGGCG

GTCAGTAGTCAAAGACGCCAACAAAATTTCACTGACAGGGAACTTTTTGACATCTTCAGA

AAGTTCGTATTCAGTAGTCAATTGCCGAGCATCAATAATGGGGATTATACCAGAAGCAAC

AGTGGAAGTCACATCTACCAACTTTGCGGTCTCAGAAAAAGCATAAACAGTTCTACTACC

GCCATTAGTGAAACTTTTCAAATCGCCCAGTGGAGAAGAAAAAGGCACAGCGATACTAGC

ATTAGCGGGCAAGGATGCAACTTTATCAACCAGGGTCCTATAGATAACCCTAGCGCCTGG

GATCATCCTTTGGACAACTCTTTCTGCCAAATCTAGGTCCAAAATCACTTCATTGATACC

ATTATTGTACAACTTGAGCAAGTTGTCGATCAGCTCCTCAAATTGGTCCTCTGTAACGGA

TGACTCAACTTGCACATTAACTTGAAGCTCAGTCGATTGAGTGAACTTGATCAGGTTGTG

CAGCTGGTCAGCAGCATAGGGAAACACGGCTTTTCCTACCAAACTCAAGGAATTATCAAA

CTCTGCAACACTTGCGTATGCAGGTAGCAAGGGAAATGTCATACTTGAAGTCGGACAGTG

AGTGTAGTCTTGAGAAATTCTGAAGCCGTATTTTTATTATCAGTGAGTCAGTCATCAGGA

GATCCTCTACGCCGGACGCATCGTGGCCGACCTGCAGGGGGGGGGGGGGCGCTGAGGTCT

GCCTCGTGAAGAAGGTGTTGCTGACTCATACCAGGCCTGAATCGCCCCATCATCCAGCCA

GAAAGTGAGGGAGCCACGGTTGATGAGAGCTTTGTTGTAGGTGGACCAGTTGGTGATTTT

GAACTTTTGCTTTGCCACGGAACGGTCTGCGTTGTCGGGAAGATGCGTGATCTGATCCTT

CAACTCAGCAAAAGTTCGATTTATTCAACAAAGCCGCCGTCCCGTCAAGTCAGCGTAATG

CTCTGCCAGTGTTACAACCAATTAACCAATTCTGATTAGAAAAACTCATCGAGCATCAAA

TGAAACTGCAATTTATTCATATCAGGATTATCAATACCATATTTTTGAAAAAGCCGTTTC

TGTAATGAAGGAGAAAACTCACCGAGGCAGTTCCATAGGATGGCAAGATCCTGGTATCGG

TCTGCGATTCCGACTCGTCCAACATCAATACAACCTATTAATTTCCCCTCGTCAAAAATAAGGTTATCAAGTGAGAAATCACCATGAGTGACGACTGAATCCGGTGAGAATGGCAAAAGC

TTATGCATTTCTTTCCAGACTTGTTCAACAGGCCAGCCATTACGCTCGTCATCAAAATCA

CTCGCATCAACCAAACCGTTATTCATTCGTGATTGCGCCTGAGCGAGACGAAATACGCGA

TCGCTGTTAAAAGGACAATTACAAACAGGAATCGAATGCAACCGGCGCAGGAACACTGCC

AGCGCATCAACAATATTTTCACCTGAATCAGGATATTCTTCTAATACCTGGAATGCTGTT

TTCCCGGGGATCGCAGTGGTGAGTAACCATGCATCATCAGGAGTACGGATAAAATGCTTG

ATGGTCGGAAGAGGCATAAATTCCGTCAGCCAGTTTAGTCTGACCATCTCATCTGTAACA

TCATTGGCAACGCTACCTTTGCCATGTTTCAGAAACAACTCTGGCGCATCGGGCTTCCCA

TACAATCGATAGATTGTCGCACCTGATTGCCCGACATTATCGCGAGCCCATTTATACCCA

TATAAATCAGCATCCATGTTGGAATTTAATCGCGGCCTCGAGCAAGACGTTTCCCGTTGA

ATATGGCTCATAACACCCCTTGTATTACTGTTTATGTAAGCAGACAGTTTTATTGTTCAT

GATGATATATTTTTATCTTGTGCAATGTAACATCAGAGATTTTGAGACACAACGTGGCTT

TCCCCCCCCCCCCTGCAGGTCGGCATCACCGGCGCCACAGGTGCGGTTGCTGGCGCCTAT

ATCGCCGACATCACCGATGGGGAAGATCGGGCTCGCCACTTCGGGCTCATGAGCGCTTGT

TTCGGCGTGGGTATGGTGGCAGGCCCCGTGGCCGGGGGACTGTTGGGCGCCATCTCCTTG

CATGCACCATTCCTTGCGGCGGCGGTGCTCAACGGCCTCAACCTACTACTGGGCTGCTTC

CTAATGCAGGAGTCGCATAAGGGAGAGCGTCGAGTATCTATGATTGGAAGTATGGGAATG

GTGATACCCGCATTCTTCAGTGTCTTGAGGTCTCCTATCAGATTATGCCCAACTAAAGCA

ACCGGAGGAGGAGATTTCATGGTAAATTTCTCTGACTTTTGGTCATCAGTAGACTCGAAC

TGTGAGACTATCTCGGTTATGACAGCAGAAATGTCCTTCTTGGAGACAGTAAATGAAGTC

CCACCAATAAAGAAATCCTTGTTATCAGGAACAAACTTCTTGTTTCGAACTTTTTCGGTG

CCTTGAACTATAAAATGTAGAGTGGATATGTCGGGTAGGAATGGAGCGGGCAAATGCTTA

CCTTCTGGACCTTCAAGAGGTATGTAGGGTTTGTAGATACTGATGCCAACTTCAGTGACA

ACGTTGCTATTTCGTTCAAACCATTCCGAATCCAGAGAAATCAAAGTTGTTTGTCTACTA

TTGATCCAAGCCAGTGCGGTCTTGAAACTGACAATAGTGTGCTCGTGTTTTGAGGTCATC

TTTGTATGAATAAATCTAGTCTTTGATCTAAATAATCTTGACGAGCCAAGGCGATAAATA

CCCAAATCTAAAACTCTTTTAAAACGTTAAAAGGACAAGTATGTCTGCCTGTATTAAACC

CCAAATCAGCTCGTAGTCTGATCCTCATCAACTTGAGGGGCACTATCTTGTTTTAGAGAA

ATTTGCGGAGATGCGATATCGAGAAAAAGGTACGCTGATTTTAAACGTGAAATTTATCTC

AAGATCTCTGCCTCGCGCGTTTCGGTGATGACGGTGAAAACCTCTGACACATGCAGCTCCCGGAGACGGTCACAGCTTGTCTGTAAGCGGATGCCGGGAGCAGACAAGCCCGTCAGGGCG

CGTCAGCGGGTGTTGGCGGGTGTCGGGGCGCAGCCATGACCCAGTCACGTAGCGATAGCG

GAGTGTATACTGGCTTAACTATGCGGCATCAGAGCAGATTGTACTGAGAGTGCACCATAT

GCGGTGTGAAATACCGCACAGATGCGTAAGGAGAAAATACCGCATCAGGCGCTCTTCCGC

TTCCTCGCTCACTGACTCGCTGCGCTCGGTCGTTCGGCTGCGGCGAGCGGTATCAGCTCACTCAAAGGCGGTAATACGGTTATCCACAGAATCAGGGGATAACGCAGGAAAGAACATGTG

AGCAAAAGGCCAGCAAAAGGCCAGGAACCGTAAAAAGGCCGCGTTGCTGGCGTTTTTCCA

TAGGCTCCGCCCCCCTGACGAGCATCACAAAAATCGACGCTCAAGTCAGAGGTGGCGAAA

CCCGACAGGACTATAAAGATACCAGGCGTTTCCCCCTGGAAGCTCCCTCGTGCGCTCTCC

TGTTCCGACCCTGCCGCTTACCGGATACCTGTCCGCCTTTCTCCCTTCGGGAAGCGTGGC

GCTTTCTCAATGCTCACGCTGTAGGTATCTCAGTTCGGTGTAGGTCGTTCGCTCCAAGCT

GGGCTGTGTGCACGAACCCCCCGTTCAGCCCGACCGCTGCGCCTTATCCGGTAACTATCG

TCTTGAGTCCAACCCGGTAAGACACGACTTATCGCCACTGGCAGCAGCCACTGGTAACAG

GATTAGCAGAGCGAGGTATGTAGGCGGTGCTACAGAGTTCTTGAAGTGGTGGCCTAACTA

CGGCTACACTAGAAGGACAGTATTTGGTATCTGCGCTCTGCTGAAGCCAGTTACCTTCGG

AAAAAGAGTTGGTAGCTCTTGATCCGGCAAACAAACCACCGCTGGTAGCGGTGGTTTTTT

TGTTTGCAAGCAGCAGATTACGCGCAGAAAAAAAGGATCTCAAGAAGATCCTTTGATCTT

TTCTACGGGGTCTGACGCTCAGTGGAACGAAAACTCACGTTAAGGGATTTTGGTCATGAG

ATTATCAAAAAGGATCTTCACCTAGATCCTTTTAAATTAAAAATGAAGTTTTAAATCAAT

CTAAAGTATATATGAGTAAACTTGGTCTGACAGTTACCAATGCTTAATCAGTGAGGCACC

TATCTCAGCGATCTGTCTATTTCGTTCATCCATAGTTGCCTGACTCCCCGTCGTGTAGAT

AACTACGATACGGGAGGGCTTACCATCTGGCCCCAGTGCTGCAATGATACCGCGAGACCC

ACGCTCACCGGCTCCAGATTTATCAGCAATAAACCAGCCAGCCGGAAGGGCCGAGCGCAG

AAGTGGTCCTGCAACTTTATCCGCCTCCATCCAGTCTATTAATTGTTGCCGGGAAGCTAG

AGTAAGTAGTTCGCCAGTTAATAGTTTGCGCAACGTTGTTGCCATTGCTGCAGGCATCGT

GGTGTCACGCTCGTCGTTTGGTATGGCTTCATTCAGCTCCGGTTCCCAACGATCAAGGCG

AGTTACATGATCCCCCATGTTGTGCAAAAAAGCGGTTAGCTCCTTCGGTCCTCCGATCGT

TGTCAGAAGTAAGTTGGCCGCAGTGTTATCACTCATGGTTATGGCAGCACTGCATAATTC

TCTTACTGTCATGCCATCCGTAAGATGCTTTTCTGTGACTGGTGAGTACTCAACCAAGTC

ATTCTGAGAATAGTGTATGCGGCGACCGAGTTGCTCTTGCCCGGCGTCAACACGGGATAA

TACCGCGCCACATAGCAGAACTTTAAAAGTGCTCATCATTGGAAAACGTTCTTCGGGGCG

AAAACTCTCAAGGATCTTACCGCTGTTGAGATCCAGTTCGATGTAACCCACTCGTGCACC

CAACTGATCTTCAGCATCTTTTACTTTCACCAGCGTTTCTGGGTGAGCAAAAACAGGAAG

GCAAAATGCCGCAAAAAAGGGAATAAGGGCGACACGGAAATGTTGAATACTCATACTCTT

CCTTTTTCAATATTATTGAAGCATTTATCAGGGTTATTGTCTCATGAGCGGATACATATT

TGAATGTATTTAGAAAAATAAACAAATAGGGGTTCCGCGCACATTTCCCCGAAAAGTGCC

ACCTGACGTCTAAGAAACCATTATTATCATGACATTAACCTATAAAAATAGGCGTATCAC

GAGGCCCTTTCGTCTTCAAGAATTAATTCTCATGTTTGACAGCTTATCATCGATAAGCTG

ACTCATGTTGGTATTGTGAAATAGACGCAGATCGGGAACACTGAAAAATAACAGTTATTA

TTCG
